# Deciding how to decide the correct double-lumen tube: a narrative review of methods and evidence

**DOI:** 10.1186/s44158-025-00286-3

**Published:** 2025-10-14

**Authors:** M. Rispoli, G. Calgaro, G. Strano, G. L. Rosboch, D. Massullo, F. Piccirillo, M. R. Nespoli, F. Coppolino, F. Piccioni

**Affiliations:** 1 Anesthesia and Intensive Care Unit, AO Dei Colli—Monaldi Hospital, Naples, Italy; 2 School of Anesthesia and Intensive Care, Humanitas University, Pieve Emanuele, Italy; 3 Anesthesia and Perioperative Medicine Unit, IRCCS Humanitas Research Hospital, Rozzano, Italy; 4 Anesthesia and Intensive Care, Dipartimento Di Anestesia, Rianimazione Ed Emergenze, AOU Città Della Salute E Della Scienza, Turin, Italy; 5 Division of Anesthesiology, Sant’Andrea Hospital, Faculty of Medicine and Psychology, Sapienza University of Rome, Rome, Italy; 6 Department of Woman, Child and General and Specialized Surgery, University of Campania “Luigi Vanvitelli”, Naples, Italy

**Keywords:** DLT sizing, Lung isolation, One-lung ventilation, Thoracic anaesthesia, Airway management

## Abstract

The selection of the appropriate size of a double-lumen tube (DLT) is a critical yet often underestimated aspect of thoracic anaesthesia. The present narrative review evaluates traditional and emerging methods for determining DLT size, including anthropometric formulas, chest X-rays, CT scans, and ultrasonography. Despite the prevalence of height- and gender-based predictions, mounting evidence underscores their restricted correlation with airway anatomy. Chest X-rays and CT scans have been shown to offer more accurate estimations of tracheobronchial dimensions, while ultrasound has been identified as a promising bedside tool. Recent meta-analytic evidence and technological advancements, including 3D reconstruction and AI-based modelling, may support a more personalised and safer approach. It is recommended that a pragmatic, image-guided strategy be employed to minimise airway trauma, improve lung isolation, and optimise patient outcomes.

## Introduction

In thoracic surgery, anaesthesiologists are tasked with skilfully managing the airway using a variety of techniques. A vital instrument in their toolkit is the double-lumen tube (DLT), crucial for achieving one-lung ventilation (OLV) and facilitating surgery by selectively collapsing and isolating a lung. The DLT is used to manage one-lung ventilation in 89–100% of cases [1–6].

Typically, for most thoracic procedures, a left-sided DLT is preferred, while a right-sided one is chosen for specific cases like left-sided pneumonectomy, left lung transplantation, or dealing with distorted anatomy of the left mainstem bronchus. Despite its long-standing use, determining the ideal size and insertion depth of the DLT remains a challenge.

Choosing an incorrectly sized DLT can result in airway trauma, oxygenation disruptions, and failure of lung separation during OLV. An undersized DLT may lead to distal positioning and increased cuff volume and pressure, raising the risk of mucosal damage or bronchial rupture. Conversely, an oversized DLT can cause airway or tracheal trauma or difficulties in entering the main bronchus [7].

Most anaesthesiologists base their DLT choice on patient height and gender [8], while others rely on chest X-rays or computed tomography to measure tracheal or bronchial dimensions. Ultrasonography emerges as a promising method for accurately determining outer tracheal width, offering further insights into left mainstem bronchial diameter and aiding in the selection of the appropriate DLT size [9].

In this narrative review, our objective is to address the challenges associated with selecting the correct DLT size. We aim to achieve this by identifying the most recent recommendations to assist in making prompt and informed decisions.

## Methods

It was decided that the principal focus of this review would be on methods described in literature to select DLT size before surgery. The literature research was conducted through PubMed, Google Scholar, and two registers (ClinicalTrials.gov and ResearchRegistry.com) between 20th and 21 st January 2025. For the literature search, studies reporting clinical methods described for DLT size selection were searched. No filters were set on the years of publication of the studies. Following the elimination of redundant records, the articles were subjected to a screening process by two authors (GC and GS). Editorials, comments on other articles, case reports, conference abstracts, reviews, study protocols, and research not focused on DLT size-selection methods were excluded from the analysis. The search strategies and the PRISMA flowchart are available in the Appendix. A shared online folder was used to make articles available to all group members. The analysis of the selected literature and the development of the manuscript were divided according to the types of DLT selection methods: anthropometric approach, chest X-ray, CT scan, ultrasound, and models based on automatic systems and artificial intelligence.

Due to the narrative nature of this review and the predominance of observational studies, we did not apply formal evidence grading systems, such as GRADE or OCEBM, to each study individually. Instead, we took a pragmatic approach. The overall strength of the evidence was summarised at the level of each methodological category: anthropometry, chest X-ray, CT scan, ultrasound, and AI-based models. We applied a simplified classification adapted from established frameworks, with the sole aim of providing clinicians with an intuitive synthesis of the heterogeneous literature (Table 1). This approach is not intended to replace formal evidence grading, but rather to serve as a transparent and practical tool that highlights the relative strengths and weaknesses of different methods. The evaluation of evidence quality was proposed as a consensus by two authors (MR, FP), shared, and discussed with all authors.

**Table 1:** Definitions of evidence levels

Evidence level	Definition
High	Evidence from well-designed prospective studies or randomised controlled trials (RCTs), with consistent results directly applicable to clinical practice
Moderate–high	Evidence from multiple perspective observational studies or small RCTs, showing consistent and reproducible results
Moderate	Evidence from observational studies (retrospective or prospective) with large samples and relatively consistent results, but potential biases or limited generalisability
Low	Evidence from small or retrospective observational studies, with weak correlations, heterogeneous results, or poor reproducibility
Very low	Preliminary or feasibility evidence, based on pilot studies, case series, or experimental data; insufficient to support clinical recommendations

### Anthropometric approach

DLT need to conform to the airway anatomy, so demographic parameters (gender and age) or anthropometric parameters (height and weight) have always been considered relevant for choosing the most suitable device for the patient. Although the evaluation of gender and anthropometric parameters is currently still widespread, more promising recent techniques have been the subject of interest in the scientific literature. One of the first studies that correlated height, gender, and left main bronchial diameter was done by Hannallah et al. [10] They demonstrated a weak correlation between height and the diameter of the left main bronchus in males, but this correlation was not verified in women. A direct correlation between gender, height, and DLT size was proposed by Martin et al. as well [11]. They reported that for women, a height of 160 cm represented the limit in the choice between 35 Fr (if shorter) and 37 Fr (if taller), with 32 Fr for patients less than 150 cm. For men, this limit was 170 cm, with 41 Fr for taller patients and 39 Fr if shorter, using 37 Fr for patients less than 160 cm. Brodsky and Lemmens, in a study of 192 patients, confirmed a weak but significant correlation between age, height, and left bronchial width (LBW) in men, and only for height and LBW in women [12]. Duthie allocated DLT according to patients’ height, with specific sizes for different heights and genders, reporting proper positioning in 96.3% of their patients [13].

The anthropometric approach remains widely applied in clinical practice despite weak evidence, as evidenced by an Italian retrospective multicentre study of 909 patients [14]. Moreover, in a retrospective study involving 179 Asian patients, Ideris et al. reported a poor correlation between DLT size with height or weight for both genders, indicating a need for downsizing in many cases [15].

Notwithstanding the dearth of compelling evidence, the choice of DLT size based on anthropometry is recommended in several papers on thoracic anaesthesia. This is likely due more to the inevitable need to provide an objective reference point for selecting the DLT size rather than the strength of the scientific rationale. Another challenging aspect is that not all studies evaluate clinical outcomes based on the choice of different tube sizes; instead, they often focus on the correspondence between the DLT size and the patient’s radiological anatomy. For example, Amar conducted a prospective study involving 300 patients using a 35 Fr DLT regardless of gender and/or height [16]. They found a similar incidence of intraoperative hypoxemia, lung isolation failure, or the need for DLT repositioning during surgery compared to the standard approach of “choosing the largest possible DLT”. Similar findings were reported by Ellensohn et al., who highlighted the significant variability in lower airway dimensions among adults and the lack of correlation between these measurements and the patient’s height or weight [17]. Current recommendations for selecting DLT sizes based on height and gender carry the risk of dimensional mismatch, which may result in airway injury or inadequate lung isolation. This mismatch is especially pronounced in females, with incompatibility rates in the left distal mainstem bronchus reaching as high as 50%.

### Chest X-ray approach

In the 1990s, some authors proposed using radiological images to improve the accuracy of choosing the appropriate size for a left DLT. Hannallah et al. attempted to measure the width of the left bronchus from chest radiographs, but measurements could be obtained for only half of the patients [10]. This approach had another limitation: the measured diameter needed to be reduced by 8–10% to account for the magnification of intrathoracic structures on the posterior-anterior radiographic film.

Based on a cadaver study that reported the diameter of the left bronchus as 68% of that of the tracheal diameter [18], Brodsky et al. conducted an observational study in 1996 on 70 patients to explore DLT size using the tracheal diameter measurement from preoperative X-rays [19]. They measured the width of the patients’ trachea at the level of the clavicles from a posterior-anterior chest radiograph, choosing the DLT size as follows: tracheal width ≥18 mm for DLT size 41 Fr, ≥16 mm for DLT size 39 Fr, ≥15 mm for DLT size 37 Fr, and ≥14 mm for DLT size 35 Fr. They encountered minor difficulty advancing the DLT through the glottis in 14.3% of patients and mild resistance while advancing the tube into the bronchus in 27.1% of patients. Nevertheless, all devices were successfully placed and functioned properly during surgery. The following year, the same group reported that this method had been effective in 487 patients, with a prevalence of 41 Fr DLT in men and 39 Fr DLT in women [20]. Chow et al. tested the reliability of this method in a cohort of 66 Asian patients, finding a positive predictive value of 77.3% and 45.5% for men and women, respectively [21]. The authors attributed the lower positive predictive value in women to anthropometric differences between Brodsky’s sample and their own (the females in the Asian study were lighter and shorter).

In a large study of 1170 patients published in 2003, Brodsky and Lemmens reported that the left DLT size choice based on the trachea width measured by chest X-ray was successful in 97% of cases (818/843) [22]. The same authors later reported that tracheal width measured on chest X-ray is a better predictor than anthropometric data (sex, height, and weight) for estimating the size of the left main bronchus [12]. Moreover, different radiological studies have derived various formulas for estimating DLT size (Table 2).

**Table 2:** Formulas based on X-ray chest for estimating DLT size

Author	Formula for calculation of LBW	Examples of result (considering a 60 years patient, 65 kg, 170 cm and TW = 18 mm)
Brodsky 2005 [12]	LBWmm = (0.45) (TWmm(CXR)) + 3.3	11.40 mm ⇒ 39 Ch
Ideris 2017 [15]	LBWmm = (0.3) (TWmm(CXR)) + 6.58 for males LBWmm = (0.49) (TWmm(CXR)) + 3.13 for females	11.98 mm ⇒ 39 Ch 11.95 mm ⇒ 39 Ch
Suvvari 2019 [23]	DLT size = 0.044 (age in years) + 0.09 (height in cm) + 0.291 (TWmm(CXR)) + 13.082	36 Ch

*LBW* Left bronchial width, *TW *Tracheal width, *CXR* Chest X-ray, *DLT* Double-lumen tube

After calculating the diameter of the LBW, the size of the DLT was assigned according to the guidelines of the study by Brodsky et al. [19]

Recently, in a retrospective study of 231 thoracic surgery patients, Chang et al. combined demographic and tracheal diameter measurements obtained from chest X-rays to develop a model to predict DLT size [24]. This model used a limited set of simple parameters (sex, height, weight, chest circumference, and tracheal diameter) to accurately predict both DLT size (*r* = 0.82) and insertion depth (*r* = 0.91). This model offers a practical tool for clinical decision-making.

### CT scan approach

The CT scan offers accurate information on the anatomy of the airways, allowing for a more precise choice of the correct DLT size. Nowadays, the CT scan is a widely used method that is easily and quickly accessible. Compared to chest X-rays, it guarantees precise measurements of the trachea and main bronchi, as well as the possibility of performing a morphological evaluation of the airways with 3D reconstruction. This capability enables the detection of abnormalities and anatomical alterations that could affect the choice of optimal tools for proper one-lung ventilation and avoid serious complications such as tracheal and bronchial injuries [23].

As early as a 1997 study [25], the CT scan was indicated as an objectively useful method for guiding the correct choice of DLT, offering the possibility of a specific choice for the individual patient through 3D reconstructions. Subsequent studies have confirmed that the use of CT scan images, even with 3D reconstruction, significantly improved the selection of the correct size of the double-lumen tube compared to using anthropometric measurements, allowing better pulmonary separation in 92.7% of cases [26].

It has been shown that measuring the diameter of the left main bronchus using the CT scan method accurately predicts the correct DLT size, particularly for patients of short stature who require a smaller DLT. In a study of 50 patients, 68% were predicted to require a smaller DLT, and six female patients were correctly predicted to need a 32 Fr DLT, significantly smaller than the 35 Fr DLT normally the smallest DLT recommended for adults [27]. Finally, in a study of Asian women, the combined CT scan measurement of the left main bronchus and cricoid ring diameters significantly improved the accuracy of DLT selection compared to using the left main bronchus diameter alone [28]. In 2005, Jeon et al. conducted an observational study of 105 patients, measuring the anteroposterior and mediolateral diameters of the left bronchus from CT and chest X-ray images, respectively [29]. The selection of DLT size was informed by the amalgamation of two measurements, ensuring that the mean of the two bronchial diameters was 0 to 2.0 mm larger than the upper limit of the 95% confidence interval of the averaged OD of the bronchial tube of the designated DLT. The authors did not report the separate results of the two methods; however, they found that in 54.3% of cases the two measurements did not agree on the choice of DLT. The study’s findings indicated that the combined approach of utilising multiple measurements ensured enhanced accuracy compared to the use of a single measurement. This finding aligns with the 2006 study by Olivier et al., which examined CT scans of 205 patients [30]. The study demonstrated that the left bronchus is invariably elliptical in shape and that the smallest diameter does not always coincide with the anteroposterior or transverse axis. Finally, it should be noted that multi-planar CT scans, which allow for 3D reconstruction of the tracheobronchial tree, reveal in most cases higher anteroposterior measurements and lower mediolateral measurements than those estimated from 2D images [31]. This finding describes a more regular and cylindrical shape of both the right and left main bronchi.

More recently, Mihatsch et al. conducted a prospective observational study in 100 patients undergoing thoracic surgery with left-sided double-lumen tubes [32]. The investigation revealed that conventional selection methods, predicated on demographics, chest X-ray, and 2D TC imaging, proved ineffective in accurately predicting left bronchial diameter in comparison to 3D bronchial measurement. These methods exhibited broad prediction intervals and a tendency towards overestimation in female subjects. A significant proportion of the study’s participants did not meet the ≥1 mm safety margin (difference between the patient’s bronchial diameter and the DLT’s outer bronchial diameter, as suggested by Hannallah et al. [10]). Specifically, 42% of women and 7% of men did not meet this criterion. The authors conclude that 3D reconstruction provides more reliable measurements and may reduce the risk of bronchial injury, particularly in high-risk female patients.

Considering the accessibility and common use of thoracic CT scan in patients undergoing thoracic surgery, along with evidence demonstrating its effectiveness in optimising DLT choice and positioning while reducing the risk of complications, it seems desirable to systematically use CT scans as a guide for selecting the correct DLT size. This approach should include measuring both the left main bronchus and cricoid ring diameters, with 3D reconstructions reserved for the most complex cases.

### New era: ultrasound (US)

As highlighted in the introduction, selecting the appropriate size for a DLT demands careful consideration due to the varied complications associated with both oversized and undersized tubes.

Preoperatively, US offers a swift assessment of the superior airway anatomy, a validation supported by CT and MRI for evaluating the subglottic airway [33]. Notably, a correlation also exists between the outer tracheal diameter measured by US just above the sternoclavicular junction and the left bronchial diameter measured by CT [9]. Moreover, previous studies have demonstrated that measuring the subglottic airway diameter using US assists in selecting appropriately sized endotracheal tubes for paediatric patients [34].

The tracheal diameter is usually measured using an ultrasound probe with a frequency of 5 to 10 MHz. The probe is placed perpendicular to the neck, just above the sternoclavicular junction, in a transverse section. The measurement is taken considering the outer diameter (in millimetres).

A study by Roldi et al. showed that using US measurements of the outer tracheal diameter alongside standard criteria (sex and height) increased the percentage of appropriately sized left DLTs from 39.2% to 86%, compared to using anthropometric data alone [9].

Šustić et al. found a robust correlation between tracheal width measured by ultrasound and tracheal and left main bronchus widths measured by CT (*r* = 0.882—*p* < 0.001 and *r* = 0.832—*p* < 0.001, respectively) [33]. Utilising ultrasound-measured tracheal width to guide LDLT size selection in 75% of cases yielded and a 95% satisfaction rate for intraoperative lung collapse. Moreover, Liu et al. also demonstrated a high correlation between ultrasonography and CT measurements of the transverse diameter of the cricoid cartilage (*r* = 0.946, *p* < 0.001) and an overall accuracy of 89% in predicting DLT size (both left and right) using US in women [35]. In a prospective observational study of 68 patients undergoing thoracic surgery, Gupta et al. compared Brodsky’s anthropometric formulas [19] with tracheal ultrasonography measurements for DLT selection [36]. The researchers found that US provided significantly higher positive predictive value (89.7% vs. 57.1%), accuracy (79.4% vs. 41.2%), faster confirmation of lung isolation, and greater surgeon satisfaction. Similarly, in another randomised comparative trial of 120 thoracic surgery patients, Mathew et al. evaluated DLT sizing based on cricoid cartilage diameter measured by US, CT, or conventional demographic methods [37]. The researchers found that both ultrasound and CT significantly improved accuracy (95% and 97.5% vs. 75%), lung collapse quality, and reduced sore throat compared to height- and gender-based selection. These findings support the hypothesis that US is superior to clinical methods.

Ultrasound presents numerous advantages over alternative imaging techniques for critically ill or emergency patients, given its wide availability, portability, repeatability, relative affordability, painlessness, and safety. Finally, considering the absence of a universal consensus in tube selection, limited access to thorax CT scans, the occasional invisibility of the left main bronchus on CXRs in 50% of patients, and the unreliable prediction of left main bronchus diameter based on anthropometric characteristics, the integration of these data with US tracheal diameter could significantly enhanced the accuracy of tube selection, mitigating both oversized and undersized LDLTs.

### Artificial intelligence and automation-based approaches

Recent developments in artificial intelligence (AI) and automated imaging analysis have opened new perspectives in airway measurement and double-lumen tube (DLT) selection [38]. AI-based algorithms, including convolutional neural networks (CNNs), can process CT images to automatically identify and measure tracheal and bronchial diameters, potentially improving precision and reducing operator dependence. Chu et al. showed that a deep learning model attained remarkably precise and expeditious airway segmentation on chest CT scans [39]. The model demonstrated a mean Dice coefficient of 0.92 and sensitivity of 0.96, thereby demonstrating a substantial improvement in performance when compared to conventional methodologies. Furthermore, the model exhibited the capacity to delineate even diminutive bronchial branches with a reduced number of false positives. Another study demonstrated that the integration of deep learning with post-processing enhanced the segmentation of lung lobes and airways on PET/CT scans [40]. The method demonstrated a Dice coefficient of 0.97 for lobes and 0.85 for airways, significantly exceeding the performance of deep learning alone, thereby substantiating its capacity for more precise lesion localisation.

In a prospective monocentric study of 173 adult patients, tracheal and bronchial diameters were measured manually by physicians and automatically with a Thoracic Volume Computer Assisted Reading software (GE Healthcare, Chicago, IL, USA) [41]. While the intraclass correlation coefficient (ICC) indicated excellent agreement between manual and automated measurements (ICC 0.93–0.97 across sites), automated analysis was not feasible in approximately 20% of cases. This finding underscores the potential and current limitations of this approach for airway measurements.

Despite their potential, AI-based systems remain in the investigational phase, with their clinical adoption still in the preliminary stage and far from becoming a part of daily practice. Contemporary clinical challenges encompass variability in imaging protocols and constrained generalisability across diverse populations. Beyond methodological variability, key barriers also include the absence of regulatory approval, the need for strict compliance with data-protection standards (e.g. General Data Protection Regulation—GDPR or Health Insurance Portability and Accountability Act—HIPAA), and the practical challenges of integrating AI tools into existing perioperative workflows. However, as data quality and algorithm robustness improve, AI could play a transformative role in personalised airway management, complementing conventional imaging and bedside methods.

## Discussion

**Fig. 1:**
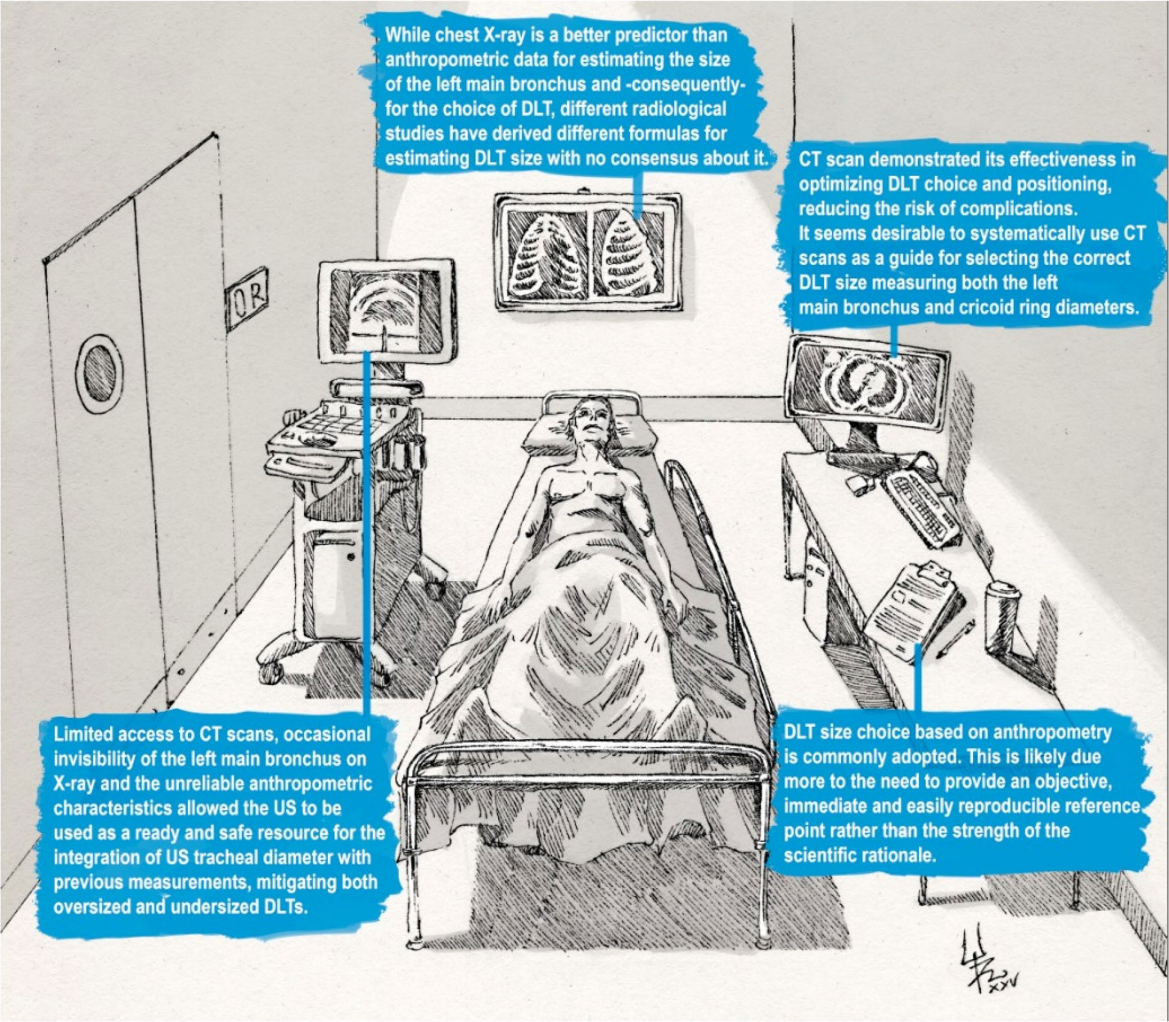
Infographic summarising the main methods used in selecting the appropriate DLT size

This review emphasises the significant variation in strategies for selecting double-lumen tubes (DLTs) as reported in the literature. Despite more than three decades of research, no universal consensus has emerged, as summarised in Table 3. Current practice often relies on local experience or institutional tradition. This variability is reflected in the broad spectrum of parameters investigated, ranging from patient anthropometry (e.g. height, weight, and sex) to radiological measurements (e.g. tracheal and bronchial diameters), as well as more recent automated and artificial intelligence (AI)-based approaches (Fig. 1). Furthermore, the literature is mainly based on observational studies, often retrospective, which do not provide robust data to determine the optimal method for selecting the DLT that combines simplicity and reliability.

**Table 3:** Summary and level of evidence of methods for DLT size selection

Method	Advantages	Limitations	Level of evidence
Anthropometry	Easy, fast, widely known and used	Poor correlation with anatomy, oversimplified	Low
Chest X-ray	More anatomical than height	50% of cases limited visibility of left main bronchus	Moderate
CT scan	Highly accurate, 3D reconstruction available	Radiation, cost, not always available. 3D reconstruction needs specific software and technical skills	Moderate–high
Ultrasound	Bedside, no radiation, portable	Operator-dependent, needs training	Moderate–high
AI/3D tools	Emerging precision tools	Limited access, not standardised	Very low

Even though it is the most widely used method today, studies based solely on anthropometric data have demonstrated limited accuracy and poor reproducibility. This confirms that height and sex alone are inadequate predictors of the optimal DLT size. Radiological assessment, particularly CT-based bronchial diameter measurement, appears to provide more reliable guidance. Using US has also proven beneficial in this context, whether on its own or combined with anthropometric data in a multimodal approach. Today, in the surgical block, the use of US is common due to locoregional anaesthesia, difficult IV access, and invasive anaesthetic procedures. Therefore, becoming more familiar with the assessment of the superior airway anatomy using US could be advantageous in emergency situations or when radiologic imaging methods are not available. So far, the lack of standardised thresholds and the variability of measurement techniques have limited its widespread use. Notably, few studies have evaluated clinical outcomes beyond tube placement, such as intraoperative complications, oxygenation, or airway trauma. This underscores a critical gap in the literature.

AI-based systems and automated airway reconstruction are promising technologies, but the current clinical experience and evidence are preliminary. The strength of these tools could be that they make measurements standardised, simple for inexperienced operators, and quick.

**Fig. 2:**
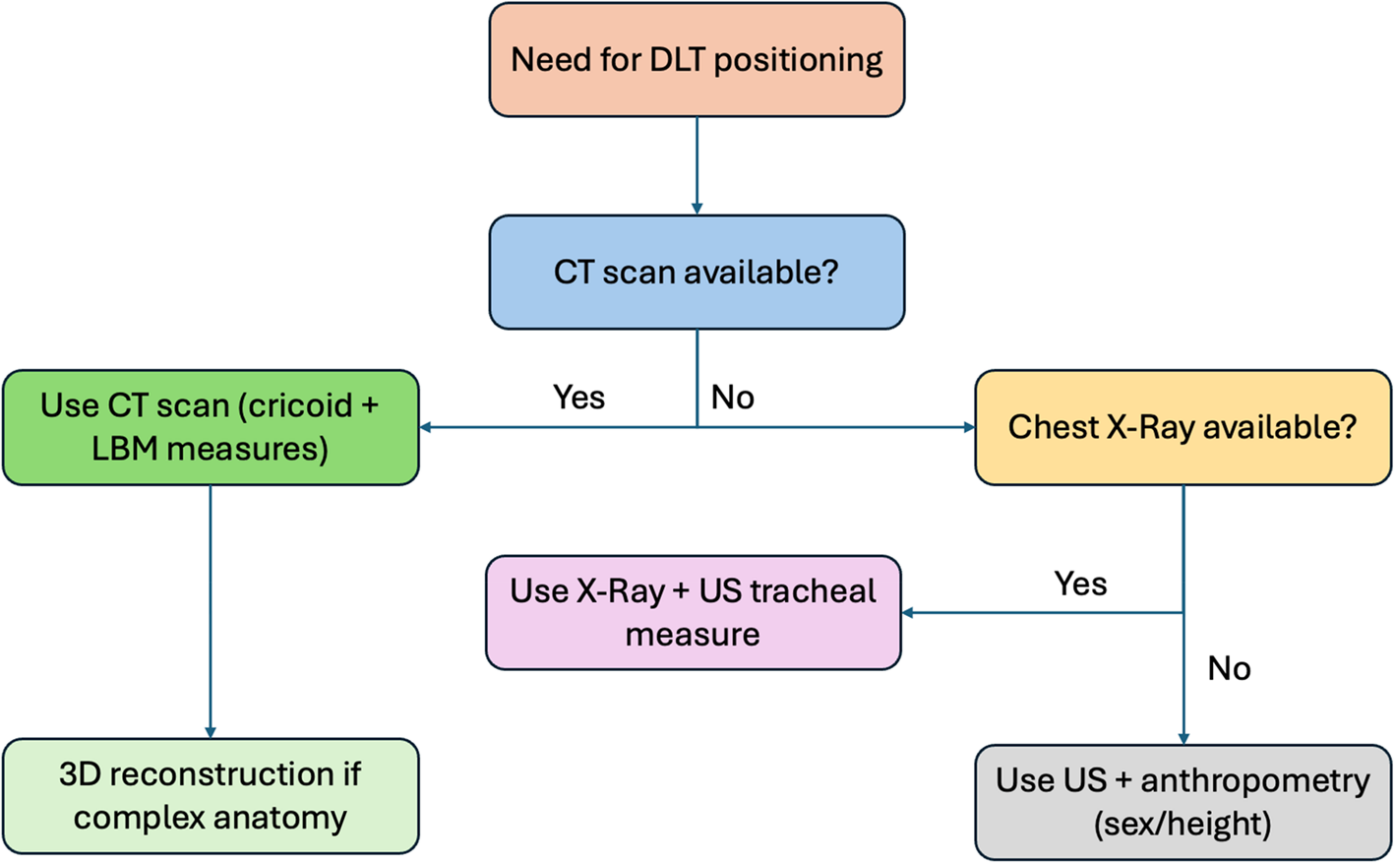
Proposed algorithm for DLT selection

Based on the current evidence, we suggest a pragmatic approach. Although anthropometric parameters can serve as an initial guide, imaging data should be considered whenever available, especially for complex thoracic procedures or patients with atypical anatomy. Furthermore, oversizing a DLT carries the risk of airway trauma and bronchial rupture, while undersizing may result in inadequate lung isolation and displacement. Thus, a structured algorithm could support balanced decision-making and reduce variability in practice. Figure 2 shows an algorithm that integrates the main strategies identified in this review into a stepwise approach. When CT or chest imaging is available, direct bronchial measurements should be prioritised, particularly for patients at higher risk for airway complications. For routine cases, sex and height can inform the initial selection of tube size, recognising their limited precision. Finally, it is important to note that intraoperative confirmation with fibreoptic bronchoscopy remains mandatory, as no preoperative method can fully guarantee the correct fit and positioning of the tube.

In conclusion, although the evidence is still fragmented and inconsistent, a structured, multimodal approach can help clinicians balance safety and efficacy when selecting a DLT size. Future research should validate standardised imaging protocols, integrate AI-assisted tools into clinical workflows, and investigate patient-centred outcomes beyond tube placement. This will lead to more consistent, evidence-based guidelines.

## Figures and Tables

**Figure:**
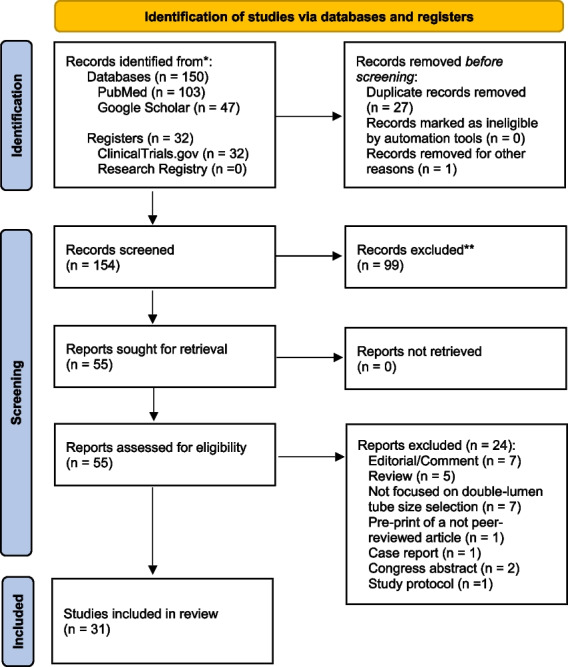
PRISMA flow diagram. *Consider, if feasible to do so, reporting the number of records identified from each database or register searched (rather than the total number across all databases/registers). **If automation tools were used, indicate how many records were excluded by a human and how many were excluded by automation tools

## Data Availability

No datasets were generated or analysed during the current study.

## Appendix

### Search strategy and keywords

#### Double-lumen tube size selection literature strategies

All searches were confined to English language papers. No restrictions on date were applied.

PubMed:

- ((double-lumen tube[Title/Abstract]) OR (double lumen tube[Title/Abstract])) AND ((size[Title/Abstract]) OR (selection[Title/Abstract]) OR (choice[Title/Abstract])) NOT ((bronchial blocker[Title/Abstract]) OR (bronchial blockers[Title/Abstract])) NOT ((paediatric[Title/Abstract]) OR (paediatric[Title/Abstract]) OR (children[Title/Abstract]) OR (infants[Title/Abstract]))
- (One-Lung Ventilation[MeSH Terms]) AND (double lumen tube[Title/Abstract]) OR (double-lumen tube[Title/Abstract]) AND ((size[Title/Abstract]) OR (selection[Title/Abstract]) (choice[Title/Abstract]))
- (Thoracic Surgery [MeSH Terms]) AND (double lumen tube[Title/Abstract]) OR (double-lumen tube[Title/Abstract]) AND ((size[Title/Abstract]) OR (selection[Title/Abstract]) (choice[Title/Abstract]))
- (Anaesthesia, Endotracheal[MeSH Terms]) AND (double lumen tube[Title/Abstract]) OR (double-lumen tube[Title/Abstract]) AND ((size[Title/Abstract]) OR (selection[Title/Abstract]) (choice[Title/Abstract]))
- (Bronchial [MeSH Terms]) AND (double lumen tube[Title/Abstract]) OR (double-lumen tube[Title/Abstract]) AND ((size[Title/Abstract]) OR (selection[Title/Abstract]) (choice[Title/Abstract]))
- (Anaesthesia, Endotracheal[MeSH Terms]) AND (double lumen tube[Title/Abstract]) OR (double-lumen tube[Title/Abstract]) AND ((size[Title/Abstract]) OR (selection[Title/Abstract]) (choice[Title/Abstract]))
- ((machine learning[Title/Abstract]) OR (artificial intelligence[Title/Abstract]) OR (automated[Title/Abstract]) AND ((double lumen tube[Title/Abstract]) OR (double-lumen tube[Title/abstract]))

Google Scholar (through Harzing’s Publish or Perish software [macOS GUI edition]):

- (double lumen tube) OR (double-lumen tube) AND (size) [Title words]
- (double lumen tube) OR (double-lumen tube) AND (selection) [Title words]
- (double lumen tube) OR (double-lumen tube) AND (choice) [Title words]

ClinicalTrials.gov:

(Double lumen tube OR Double-lumen tube) AND (size OR selection criteria OR choice)

Research Registry:

(Double lumen tube OR Double-lumen tube) AND (size)
